# Photocatalytic Degradation of Rhodamine-B and Water Densification via Eco-Friendly Synthesized Cr_2_O_3_ and Ag@Cr_2_O_3_ Using Garlic Peel Aqueous Extract

**DOI:** 10.3390/nano14030289

**Published:** 2024-01-31

**Authors:** Laila S. Alqarni, Maha D. Alghamdi, Aisha A. Alshahrani, Nasser F. Alotaibi, Shaima M. N. Moustafa, Khulaif Ashammari, Ibtihal A. Alruwaili, Amr Mohammad Nassar

**Affiliations:** 1 Chemistry Department, College of Science, Imam Mohammad Ibn Saud Islamic University (IMSIU), P.O. Box 90950, Riyadh 11623, Saudi Arabia; lassalqarni@imamu.edu.sa; 2 Department of Chemistry, Faculty of Science, Al-Baha University, P.O. Box 1988, Al-Baha 65799, Saudi Arabia; mahaalghamdi@bu.edu.sa (M.D.A.); aalshahrani@bu.edu.sa (A.A.A.); 3 Chemistry Department, College of Science, Jouf University, Sakaka 2014, Saudi Arabia; nfalotaibi@ju.edu.sa (N.F.A.); ebtehalafet98@gmail.com (I.A.A.); 4 Biology Department, College of Science, Jouf University, Sakaka 2014, Saudi Arabia; shymaa.nabil@ju.edu.sa; 5 Physics Department, College of Science, Jouf University, Sakaka 2014, Saudi Arabia; knnalshammari@ju.edu.sa

**Keywords:** biosynthesis, *Allium sativum*, Cr_2_O_3_-NPs, silver, photocatalyst, antimicrobial

## Abstract

The purification and densification of wastewater play an important role in water recycling, especially if the materials used in water recycling are other types of recycled waste. Therefore, considering this view in this study, the biosynthesis of silver-decorated chromium oxide nanoparticles utilizing a wasted *Allium sativum* (garlic) peel extract is investigated. The aqueous extract of garlic peel (GPE) was treated with silver nitrate, chromium nitrate, and a mixture of silver nitrate and chromium nitrate to synthesize silver nanoparticles (Ag-garlic), chromium oxide nanoparticles (Cr_2_O_3_-garlic), and silver-decorated chromium oxide nanoparticles (Ag@Cr_2_O_3_-garlic), respectively. The synthesized nanoparticles were elucidated via thermal gravimetric analysis (TGA), infrared spectra (FT-IR), absorption spectra (UV-Vis), scanning electron microscopy (SEM), energy dispersive X-ray analysis (EDX), X-ray diffraction (XRD), X-ray photoelectron spectroscopy (XPS), and transmission electron microscopy (TEM). Antimicrobial activity studies were conducted against waterborne germs, bacterial strains (*Bacillus subtilis*, *Enterococcus faecium*, *Escherichia coli*, and *Pseudomonas aeruginosa*), and fungal strains (*Alternaria porri*, *Aspergillus flavus*, *Aspergillus niger*, *Fuserium oxysporum*, and *Trichoderma longibrachiatum*) and showed significant levels of antimicrobial activity. The results revealed that Ag@Cr_2_O_3_ significantly improved antimicrobial activity due to their synergistic effect. The photocatalytic activity of nanoparticles was assessed using Rhodamine B dye (5 ppm) under solar irradiation. Cr_2_O_3_-garlic exhibited the best activity as a photocatalyst among the studied nanoparticles, with 97.5% degradation efficiency under optimal conditions.

## 1. Introduction

Nowadays, one of the important aims of environmental research is the circular economy, which aims to increase sustainable development by producing environmentally beneficial products from waste and by-products [1,2]. It also seeks to reduce pollution from industry and humans [3]. Metal and metal oxide nanoparticles are of tremendous importance due to their unique features. The remarkable electrical, optical, magnetic, and catalytic capabilities of metal and metal oxide nanoparticles (NPs) open a wide range of applications, including industrial, medicinal, and environmental applications [4]. The use of bioresources for the green synthesis of metal and metal oxide nanoparticles is gaining popularity among researchers as a sustainable and environmental strategy [5]. The phytochemicals in plants function as a reducing and capping agent, leading to the formation of nanoparticles. Since many phytochemicals express biological activities, the resulting nanoparticles could be employed for biological and medicinal applications by utilizing them as antibacterial, antifungal, anticancer, and antioxidant agents [6,7]. Green synthesis is better than common chemical synthesis because it is less expensive, causes less pollution, and reduces environmental and human health risks. Due to their low toxicity and less detrimental effects on the environment, green-synthesizing NPs derived from plant extracts have recently gained attention for the synthesis of different metal and metal oxide nanoparticles [8,9,10,11,12,13].

Chromium (III) oxide (Cr_2_O_3_) is a semiconductor with both n-type and p-type behaviors [14] that has been widely studied in nanopowder and thin layers in several applications and has drawn a lot of attention in science and technology [15,16,17]. Recently, there have been several methods for the synthesis of Cr_2_O_3_ nanoparticles using plant extracts [18,19], fungal extracts [20], and other green sources [21]. To the best of our knowledge, there is no report in the literature describing the synthesis of Cr_2_O_3_-NPs via a garlic peel extract. Silver nanoparticles (Ag-NPs) are attracting increasing interest in the research community due to their various effective applications. Their applications include biological, biomedical, optical, thermal, catalytic, electrochemical, and electrical applications [22,23]. There have been a variety of green strategies to prepare Ag-NPs [24]. The Ag@Cr_2_O_3_ nanocomposite has been synthesized using different methods [25,26,27,28]. For the enhancement of the properties of Cr_2_O_3_-NPs in their applications, Ag-NPs were added to form Ag/Cr_2_O_3_, as discussed by Cao. J et al. [29]. This research looked at how Ag/Cr_2_O_3_ increased sensitivity to triethylamine gas when compared to Cr_2_O_3_. After careful research in the literature, it is clear that Ag@Cr_2_O_3_ has not been synthesized via green methods.

The discovery of safe and eco-friendly antimicrobial agents still plays an important role in the treatment of several environmental issues. The development of eco-friendly processes for synthesizing nanoparticles could, therefore, contribute to finding a solution to this concern. Although the antimicrobial activity of Cr_2_O_3_ has not been studied widely, these NPs are good candidates as antimicrobial agents [30]. Using *H. thebaica* extracts, Khalil et al. [31] reported the synthesis of Cr_2_O_3_NPs and studied their antimicrobial activity. According to their findings, Cr_2_O_3_ NPs significantly inhibited the growth of the bacteria *E. coli*, *B. subtilis*, *S. epidermidis*, *K. pneumonia*, and *P. aeruginosa*, as well as the fungi *A. niger* and *F. solani*, *Mucor* sp., and *A. fumigatus.* The antimicrobial activity of Cr_2_O_3_-NPs synthesized using the *Callostemon viminalis* flower extract was reported by Hassan et al. [32]. They revealed that biosynthesized Cr_2_O_3_NPs exhibited remarkable antimicrobial activity. *Apis mellifera* was used to make chromium oxide nanoparticles, Cr_2_O_3_NPs, which show remarkable antibacterial activity [33].

In the current work, a novel green approach for the synthesis of Cr_2_O_3_ and Ag@Cr_2_O_3_ via the wasted garlic peel extract was carried out for the valorization of food waste recycling and transformation into valuable materials for benefits in vital applications. In light of their structural, spectroscopic, and thermal properties, the characterization of nanoparticles was studied. In order to determine how garlic peel extract and its nanoparticles performed in antimicrobial studies, their capacity to inhibit microbial growth was examined. The photocatalytic activity of the biosynthesized nanoparticles was examined under solar irradiation for the degradation of rhodamine B.

## 2. Experimental Section

### 2.1. Materials

*Allium sativum* (garlic) peels were collected from a nearby Sakaka market. Sigma-Aldrich (Missouri, MO, USA) supplied the chemicals chromium nitrate, silver nitrate, and rhodamine B, which were used as received without additional purification. Using Milli-Q water, aqueous solutions were prepared.

### 2.2. Instruments

Thermo Scientific’s Quattro S instrument (Waltham, MA, USA) was employed for SEM photodetection. Fourier-transform infrared (FT-IR) measurements were made using the IRTracer-100 SHIMADZU spectrophotometer (Kyoto, Japan). Using a copper radiation source, the XRD-7000 SHIMADZU was used for investigating X-ray diffraction (XRD) patterns. XPS Amicus mono-energetic beams of X-ray photons with a higher energy than 1 keV (Manchester, UK) were used to obtain XPS spectra. The TGA-51SHIMADZU was used to detect thermal gravimetric analysis (TGA) at a heating rate of 10 °C/min. Using Labomed-Spector 99 UV-Vis double-beam 3200, UV-Vis spectra were detected. Utilizing an 80 kV operated JEOL GEM-1010 transmission electron microscope (Tokyo, Japan) with a JEOL GEM-1010 transmission electron microscope operating at 80 kV, the transmittance electron microscope (TEM) images were taken.

### 2.3. Preparation of Garlic Peels Aqueous Extract (GPE)

Garlic peels were cleaned with distilled water to remove dirt and other impurities, and they were then allowed to air-dry. To obtain the aqueous extract, 5 g of garlic peel was heated at 75 °C for 1 h in 500 mL of distilled water. The solution was preserved for further work after being filtered using Whatman’s No. 1 filter paper.

### 2.4. Green Synthesis of Nanoparticles, Cr_2_O_3_-Garlic, Ag-Garlic, and Ag@Cr_2_O_3_-Garlic

Cr_2_O_3_-garlic was synthesized as follows: A 100 mL solution of GPE was added to 100 mL of 0.1 M chromium nitrate with stirring for 1 h until a precipitate appeared at the bottom of the flask; this was then incubated in a water bath for 3 h. The precipitate was filtered with filter paper, Whatman’s No. 1, air dried and then heated in a muffle furnace at 550 °C for 2 h. The obtained solid was preserved for further characterization and antimicrobial studies. The same procedures were used for the synthesis of Ag-garlic and Ag@Cr_2_O_3_-garlic using silver nitrate and a mixture of silver nitrate/chromium nitrate, respectively.

### 2.5. Antimicrobial Assay

#### 2.5.1. Collection of Samples

Waterborne germs used in this study were as follows: bacterial strains (*Bacillus subtilis*, *Enterococcus faecium*, *Escherichia coli*, and *Pseudomonas aeruginosa*) and fungal strains (*Alternaria porri*, *Aspergillus flavus*, *Aspergillus niger*, *Fuserium oxysporum*, and *Trichoderma longibrachiatum*). All germs were isolated at biology department labs at the College of Science at Jouf University.

#### 2.5.2. Evaluation of Antimicrobial Activity

The antimicrobial characteristics were investigated using determination of minimum inhibition concentrations (MICs) and an agar well diffusion methods.

The MIC values were assessed based on the lowest concentration of garlic peels, Ag-garlic, Cr_2_O_3_-garlic, and Ag@Cr_2_O_3_-garlic, which showed a complete absence of mycelium growth. Fungal isolates were cultivated on PDA media plates at 26 ^o^C for 6 days to yield fungal inoculums. Conidia were obtained by scraping them off surfaces with a sterile spatula, and they were then suspended in sterile-distilled water. Under a light microscope, the spores were counted, and the suspension was diluted with sterile-distilled water to reach the desired concentration of (15 spores per µL). A volume of 50 µL of spore suspension was placed on a 250 mL Erlenmeyer flask containing 100 mL of a PD broth medium and supplemented with 10 mL of different concentrations of the garlic peels, Ag-garlic, Cr_2_O_3_-garlic, and Ag@Cr_2_O_3_-garlic (1, 20, 40, 60, 80, and 100 ppm). Incubation was performed at 26 °C for 7 days in the dark with continuous shaking. Each flask’s mycelial mat was filtered through filter paper, Whatman’s No. 1, and washed twice with distilled water before drying in an oven at 60 °C for 24 h. The mycelial mat of each fungus was measured by subtracting the dry filter paper’s weight from the total weight. Controls were performed using PDB media only.

The agar-well diffusion approach was utilized to ascertain the antifungal activity [34]. Potato broth was used as the inoculation medium for the isolates. A cork borer was used to construct the 0.5 mm-diameter wells on each isolate, which were prepared on separate agar plates. The samples, totaling 100 µL, were added to the well. After that, the Petri plates were incubated for seven days at 26 °C. The inhibition zone of pathogenic fungus was used to measure the antifungal activity of the samples [35].

For antibacterial activity, a broth macro-dilution technique was used to determine the MIC, which was performed by preparing tubes that contained 2 mL (of nutrient broth), 20 µL of tested bacteria, and serial dilutions yielding concentrations of 20, 25, 30, 35, 40, 45, 50, 55, and 60 ppm of the garlic peels, Ag-garlic, Cr_2_O_3_-garlic, and Ag@Cr_2_O_3_-garlic in each tube. After that, the tubes were incubated at 37 °C for 48 h.

Antibacterial activity was determined using the agar-well diffusion method. The isolates were inoculated using a Nutrient Agar medium. Each isolate was prepared onto separate agar plates and the wells (0.5 mm in diameter). The well was filled with 100 µL of the garlic peels, Ag-garlic, Cr_2_O_3_-garlic, and Ag@Cr_2_O_3_-garlic. After that, the Petri plates were incubated for 48 h at 30 °C. The inhibition zone was found in order to quantify the samples’ antibacterial activity [36].

### 2.6. Photocatalytic Studies

The synthesized nanoparticles were examined as photocatalysts using rhodamine B (RhB) dye under sunlight. In order to achieve equilibrium for RhB on the catalyst’s surface, 20 mg of the catalyst was added to 50 mL of 5 ppm RhB in a 200 mL conical flask and stirred for 10 min in the dark. The solution was then subjected to 40 × 10^3^ LUX of sunlight. Then, 3 mL of the treated solution was taken out every 10 min and centrifuged for 2 min to isolate the catalyst.

## 3. Results and Discussion

**Scheme 1:**
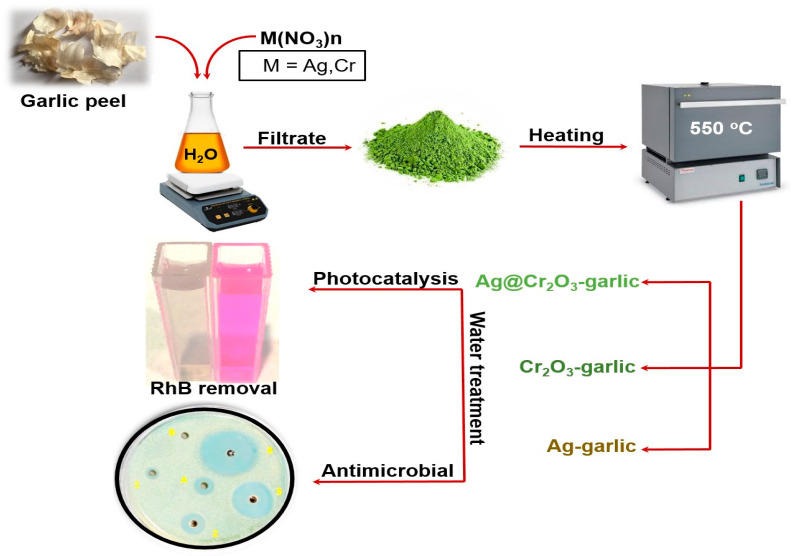
Green synthesis and applications of nanoparticles.

The aim of this work is to recycle food waste that is collected for no cost into valuable materials for use in the disinfection and purification of wastewater. Chromium oxide (Cr_2_O_3_-garlic), silver (Ag-garlic), and silver-decorated chromium oxide (Ag@Cr_2_O_3_-garlic) were biogenically synthesized using an aqueous extract of garlic peel. Bioactivity was studied against a wide spectrum of isolated water germs. The photocatalytic properties were evaluated for the decomposition of RhB dye under sunlight, Scheme 1.

### 3.1. Characterization

#### 3.1.1. XRD

**Figure 1:**
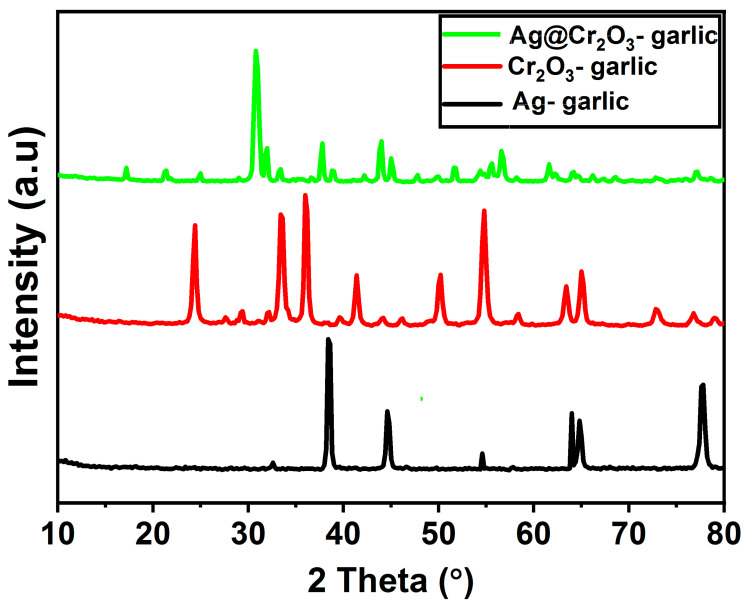
XRD of Ag-garlic, Cr_2_O_3_-garlic, and Ag@Cr_2_O_3_-garlic.

The XRD pattern of the synthesized nanoparticles on the scale of 2*θ*  =  10–80 is shown in Figure 1. The materials were successfully nanocrystalline, as indicated by the sharp and strong peaks. The Ag-garlic XRD investigation findings revealed strong diffraction peaks. The diffraction peaks present at angles 2*θ* = 77.5°, 64.6°, 44.5°, and 38.3° were perfectly matched with the (311), (220), (200), and (111) planes of silver in the metallic form (JCPDS #: 04-0783) [37]. Additionally, the diffraction pattern showed no peaks associated with AgO_2_, Ag_3_O_4_, or other Ag molecules, demonstrating the high purity of nanoparticles. The XRD of Cr_2_O_3_-garlic showed Bragg’s diffraction peaks at 2*θ* = 24.5°, 33.6°, 36.2°, 41.5°, 50.2°, 63.4°, 76.8°, and 79.1°, respectively, correlating to the crystal planes of (012), (104), (110), (113), (024), (116), (214), and (306). The diffraction peaks of Cr_2_O_3_ nanoparticles and the (JCPDS 38–1479) diffraction standard matched quite well [38]. The XRD of Ag@Cr_2_O_3_-garlic shows the shift in Ag and Cr_2_O_3_ peaks due to the involvement of silver on the surface of chromium oxide, which led to a difference in crystallinity in the formed nanocomposite [39]. The loading of silver that covers an area of Cr_2_O_3_ results in low-intensity peaks in the XRD of Ag@Cr_2_O_3_-garlic compared to Ag-garlic and Cr_2_O_3_-garlic, which have free surfaces.

#### 3.1.2. TGA

**Figure 2:**
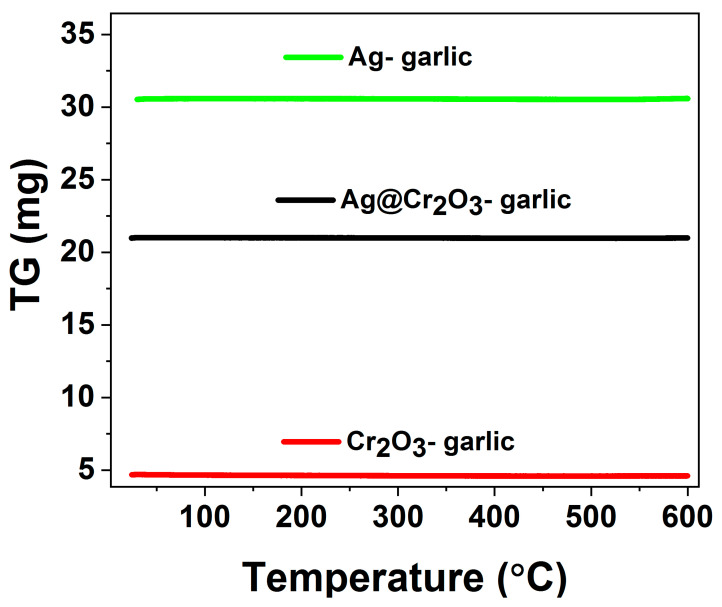
TGA of Ag-garlic, Cr_2_O_3_-garlic, and Ag@Cr_2_O_3_-garlic.

TGA of the produced NPs was achieved in the 30–600 °C range to guarantee purity and thermal stability (Figure 2). The TGA curves show no deprivation, which indicates the elimination of the bioorganic molecules from the garlic extract after calcination and confirms the purity of the biogenic synthesized NPs.

#### 3.1.3. XPS Analysis

**Figure 3:**
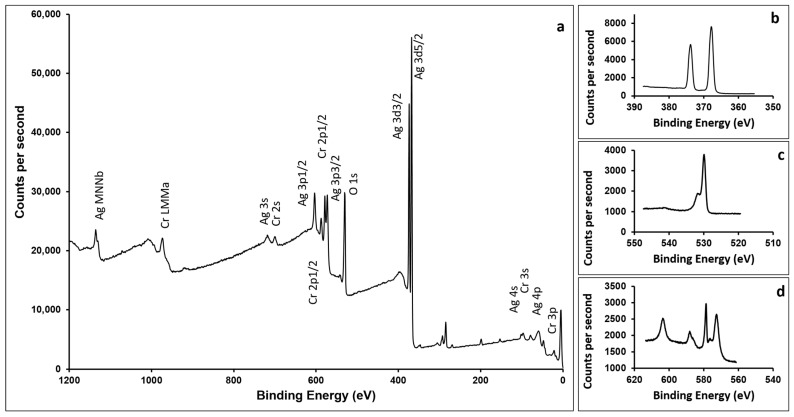
XPS of Ag@Cr_2_O_3_-garlic at full range (**a**), the corresponding expansion of Ag 3d (**b**), the corresponding expansion of O 1s (**c**), and the corresponding expansion of Cr 2p&Ag 3p (**d**).

**Figure 4:**
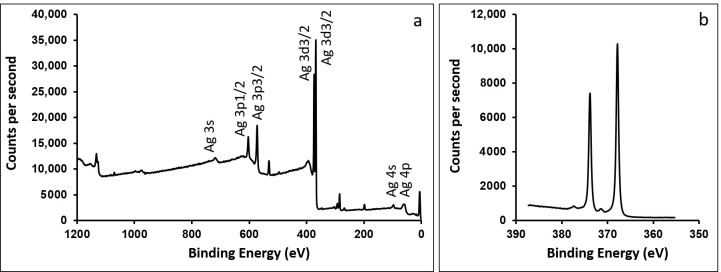
XPS of Ag-garlic at full range (**a**) and the corresponding expansion of Ag 3d (**b**).

The X-ray electron spectroscopy (XPS) results of Ag@Cr_2_O_3_-garlic and Ag-garlic are illustrated in Figure 3 and Figure 4, respectively. The components of Ag@Cr_2_O_3_-garlic are revealed in Figure 3a. As shown, the formation of Cr_2_O_3_-NPs and the presence of Ag-NPs in their decorated form can be confirmed. The spectrum shows the presence of Ag, Cr, and O as the main components of the synthesized nanocomposite. The presence of a few other components like Ca, K, and Na is due to some metallic residues in the garlic peel extract and is consistent with previous studies [40]. Figure 3b shows that the binding energies of silver are 373.90 eV and 367.90 eV, which correspond to Ag 3d_5/2_ and Ag 3d_3/2_, respectively_._ The separation between these two binding peaks is roughly 6.00 eV. This confirms how a reduction in Ag^+^ forms metallic Ag using the garlic peel extract [41]. The peak centering at 530 eV corresponds to the O1s, as seen in Figure 3c. In Figure 3d, there are two peaks centered at 576 and 586 eV, which correspond to Cr^3+^ 2p_3/2_ and Cr^3+^ 2p_1/2_, respectively, which agree well with previous reports [42]. Also, Figure 3d shows peaks due to Ag 3p observed at 573 and 604 eV, which correspond to Ag 3p_3/2_ and Ag 3p_1/2_, respectively. Figure 4a shows the components of Ag-garlic. The spectrum also exhibits the presence of a very low concentration of metallic residues (Na, K, and Ca) from the garlic peel extract. The reduction in Ag^+^ to Ag^0^ using the garlic peel extract is confirmed by the observation of two peaks at 373.85 eV and 367.85 eV, which are attributed to Ag 3d_5/2_ and Ag 3d_3/2_, respectively. The formation of Ag-NPs is demonstrated by the 6.00 eV separations between these two binding peaks [41].

#### 3.1.4. FT-IR

**Figure 5:**
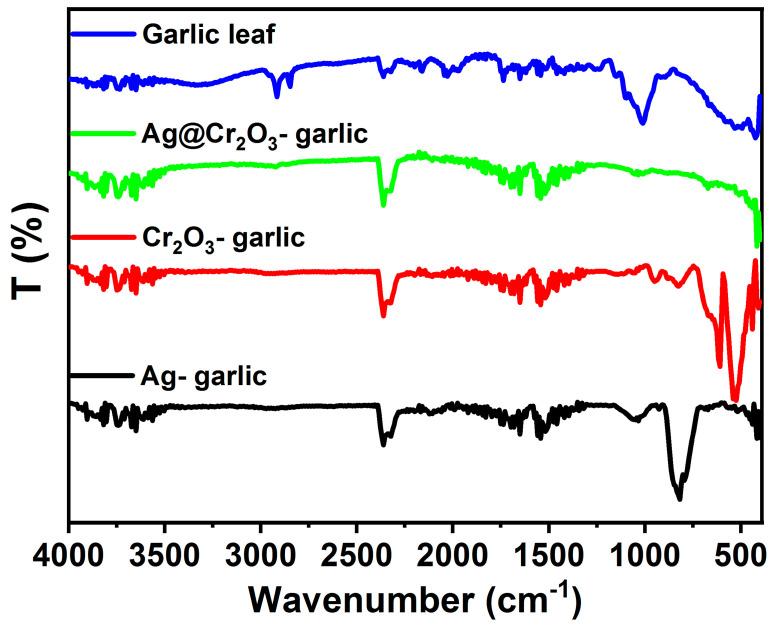
IR spectra of garlic peel, Ag-garlic, Cr_2_O_3_-garlic, and Ag@Cr_2_O_3_-garlic.

The FT-IR spectra of a solid garlic peel and synthetic NPs in the 400–4000 cm^−1^ spectral range are shown in Figure 5. A solid garlic peel spectra revealed several bands at 3350, 2900, 1710, 1660, and 1160 cm^−1^. The stretching bonds O-H, C-H, C=O, C=C, and C-O, which are present in bioorganic molecules such as phenolic compounds, amino acids, and carboxylic acid compounds, are responsible for these bands [43]. The main organosulfur ingredient in garlic is called allicin [44]. More well-defined bands connected to allicin were discovered at 800 cm^−1^, 1050 cm^−1^, and 1215 cm^−1^ attributed to v_C-S_, v_S = O_, and v_S-S_, respectively [45]. The weak bands between 400 and 600 cm^−1^ were detected in Ag-NPs and are related to metal–metal bonds [46]. The vibrational modes observed at 420, 560, and 650 cm^−1^ indicate chromium oxide mode. The strong absorption band at 560 cm^−1^ and the relatively weak absorption band at 420 cm^−1^ can both be attributed to Cr-O bonds in the bending mode, while the high absorption band at 650 cm^−1^ is reported for the existence of crystalline Cr_2_O_3_. The low-intensity band at 1050 cm^−1^ is due to Cr-O stretching vibrations [47].

#### 3.1.5. UV-Vis Spectra

**Figure 6:**
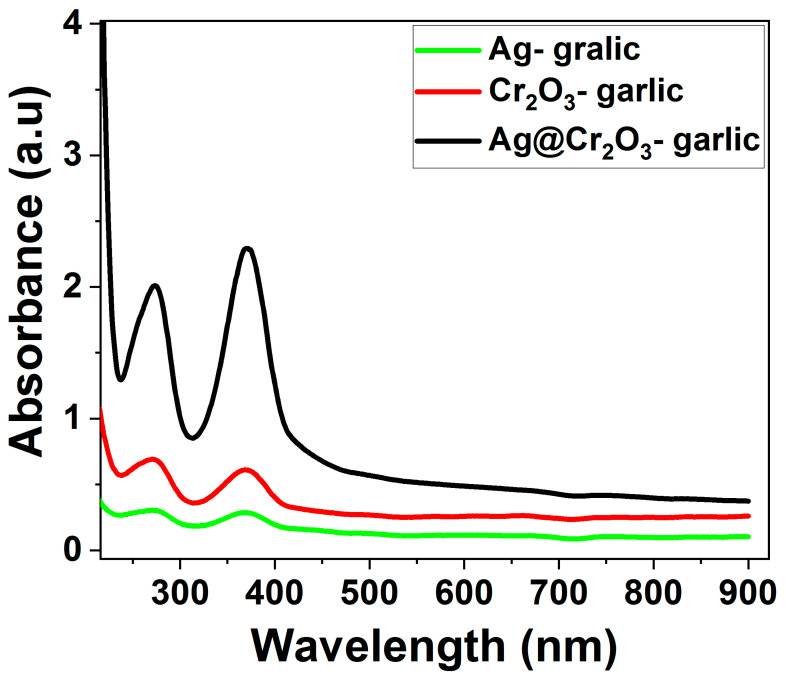
UV-Vis spectra of Ag-garlic, Cr_2_O_3_-garlic, and Ag@Cr_2_O_3_-garlic.

Figure 6 illustrates the UV-Vis spectra of the synthesized nanoparticles. The spectra of synthesized Ag-garlic, Cr_2_O_3_-garlic, and Ag@Cr_2_O_3_-garlic show two distinct surface plasmon resonances, with absorption peaks at 270 and 385 nm [48,49,50].

#### 3.1.6. SEM Studies

**Figure 7:**
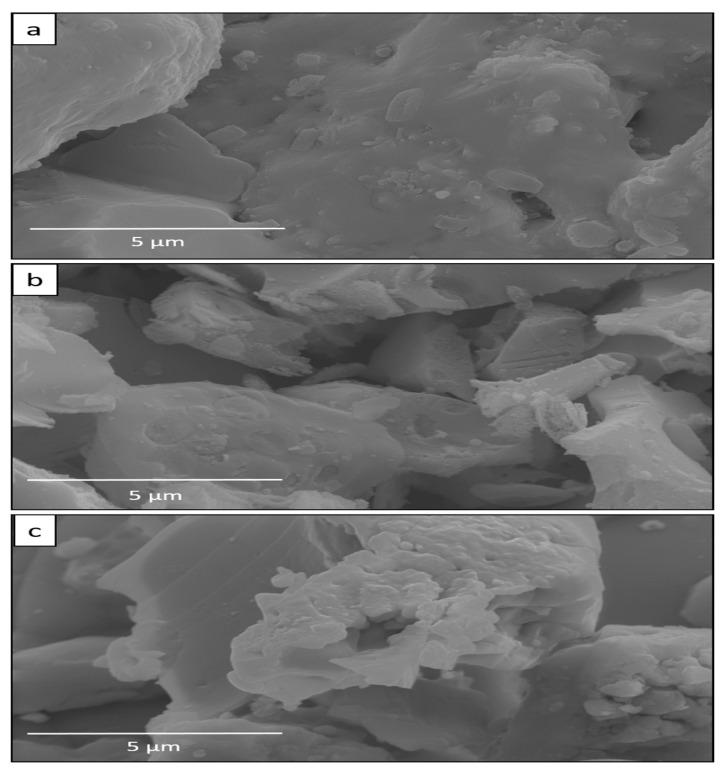
SEM images of Ag-garlic (**a**), Cr_2_O_3_-garlic (**b**), and Ag@Cr_2_O_3_-garlic (**c**).

The nanoparticles produced via GPE are examined using SEM analysis, as shown in Figure 7. The nanoparticles exhibited a lack of direct interaction with each other even inside the aggregates, suggesting that the presence of a capping agent effectively stabilizes the particles [51]. Based on SEM data, it is possible that the smaller porous particles experienced aggregation, resulting in the formation of larger ones.

#### 3.1.7. TEM Studies

**Figure 8:**
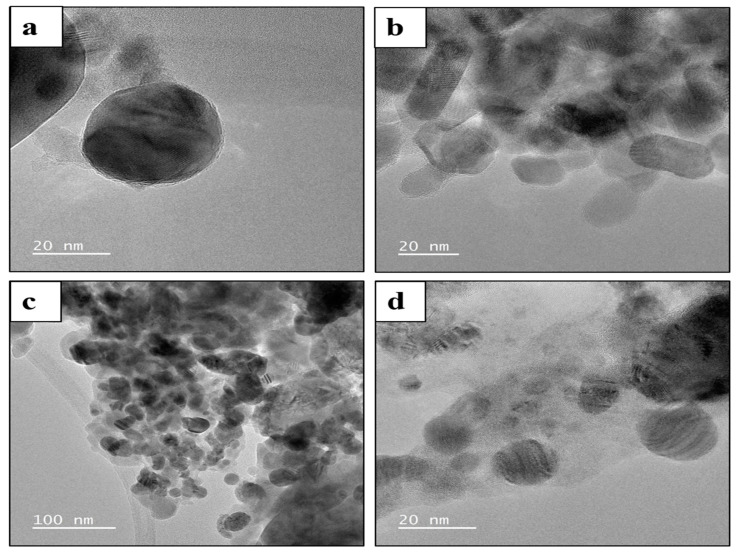
TEM images of Ag-garlic (**a**), Cr_2_O_3_-garlic (**b**), and Ag@Cr_2_O_3_-garlic (**c**,**d**).

In order to obtain insight into the formation, size, and morphology of the nanoparticles, TEM analysis was utilized, Figure 8. The formation of the synthetic nanoparticle was verified using TEM photos. TEM images indicate that Ag-garlic takes a spherical shape with a size ≈50 nm, Figure 8a. The TEM image of Cr_2_O_3_-garlic, Figure 8b, is mostly short and rod-shaped, with a 20–30 nm length. The TEM images of Ag@Cr_2_O_3_-garlic show both short, rod-shaped chromium oxide nanoparticles and spherical silver nanoparticles in decoration form, Figure 8c,d.

### 3.2. Antimicrobial Assay

**Figure 9:**
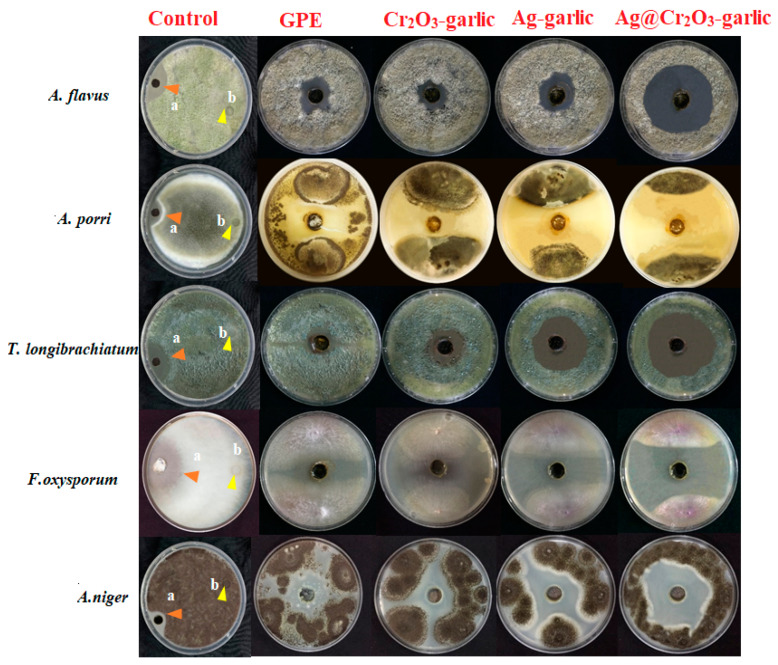
Effect of GPE and its synthesized nanoparticles on the mycelial growth of different pathogenic fungi. Controls: miconazole (a) and PDB broth media (b) on PDA at 26 °C for 6 days in the dark.

**Figure 10:**
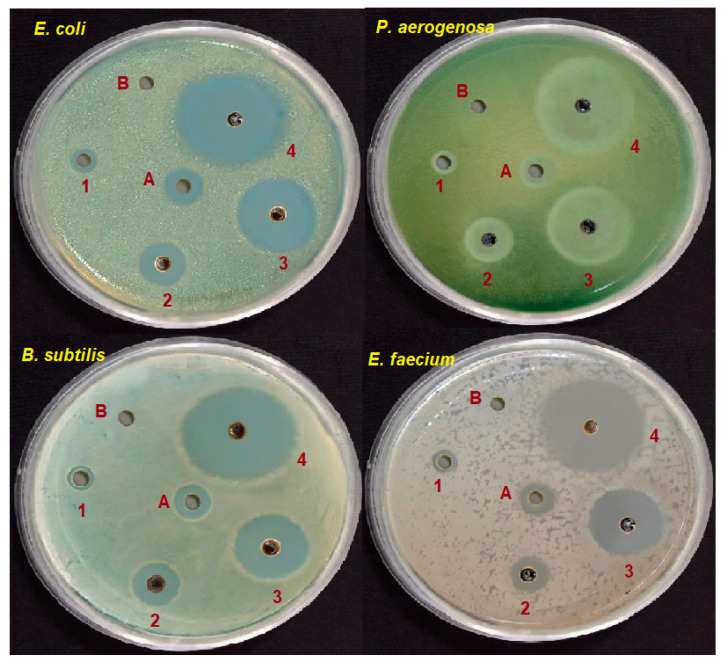
Effect of garlic peel extracts (1), Cr_2_O_3_-garlic (2), Ag-garlic (3), and Ag@Cr_2_O_3_-garlic (4) (40 ppm) on the growth of different bacterial strains. Controls: amoxicillin (A) and NB media (B) on NA at 30 °C for 2 days in the dark.

In vitro tests were performed to assess the antimicrobial susceptibility of waterborne microorganisms to the garlic extract and its biosynthesized chromium oxide nanoparticles (Cr_2_O_3_-garlic), silver oxide nanoparticles (Ag-garlic), and composites (Ag@Cr_2_O_3_-garlic) against fungal and bacterial germs. The fungal germs include *Alternaria porri*, *Aspergillus flavus*, *A. niger*, *Fuserium oxysporum*, and *Trichoderma longibrachiatum.* Four bacterial isolates were used in this study as follows: Gram-positive bacteria (*Bacillus subtilis* and *Enterococcus faecium*) and Gram-negative bacteria (*E. coli* and *P. aeruginosa*). An agar-well diffusion method, a broth macro-dilution technique, and MIC determination served as reference methods for these experiments. An agar-well diffusion method, a broth macro-dilution technique, and MIC determination served as reference methods for these experiments. The antimicrobial results are provided in Table 1 and Table 2 and illustrated in Figure 9 and Figure 10.

Except for *Fusarium*, the MIC of Ag@Cr_2_O_3_-garlic at 40 ppm was found to be the optimal concentration for inhibiting fungi growth. But when Cr_2_O_3_-garlic and Ag-garlic were used, the MIC was at 60 ppm for all tested fungi except for *Aspergillus* spp. and *Fusarium* at 80 ppm. Additionally, the MIC of the garlic extract was 80 ppm. In the case of bacteria, the results showed that the ability of Ag@Cr_2_O_3_-garlic is absolutely the best to inhibit different bacterial cells, as the MIC ranged from 25 ppm to 30 ppm, while in the case of Ag-garlic, the MIC ranged from 35 ppm to 45 ppm. The range of 50 ppm to 45 ppm is the MIC of Cr_2_O_3_-garlic. The results indicate that the MIC of the garlic extract ranges between 50 ppm and 60 ppm. As a result, we can conclude that the results were as follows: Ag@Cr_2_O_3_-garlic > Ag-garlic > Cr_2_O_3_-garlic > GPE. These results were due to the synergistic effects of the overall nanocomposite rather than the individual effects of Ag and Cr_2_O_3_.

**Figure 11:**
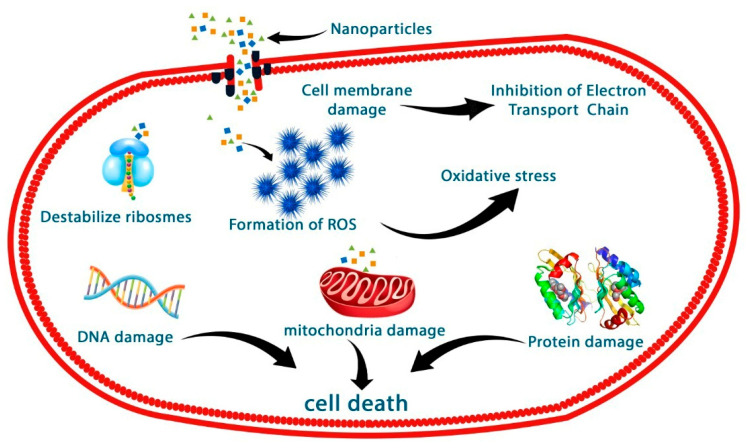
Suggested mechanism of nanoparticle activity.

The suggested mechanism can be based on the fact that the nanoparticles can enter microbial cell walls, which cause the denaturation of cell membranes and impede microbial growth, Figure 11. Then, the nanoparticles can generate reactive oxygen species (ROS), which are produced at a higher rate and prevent adenosine triphosphate (ATP) release, DNA replication, and cell growth [52]. Furthermore, as a result of cell membrane denaturation, the intracellular and extracellular components of the microbial cell membrane may break, resulting in cell lysis [53].

The antimicrobial results in this study are compared with similar studies (Table 3).

### 3.3. Photocatalytic Studies

**Figure 12:**
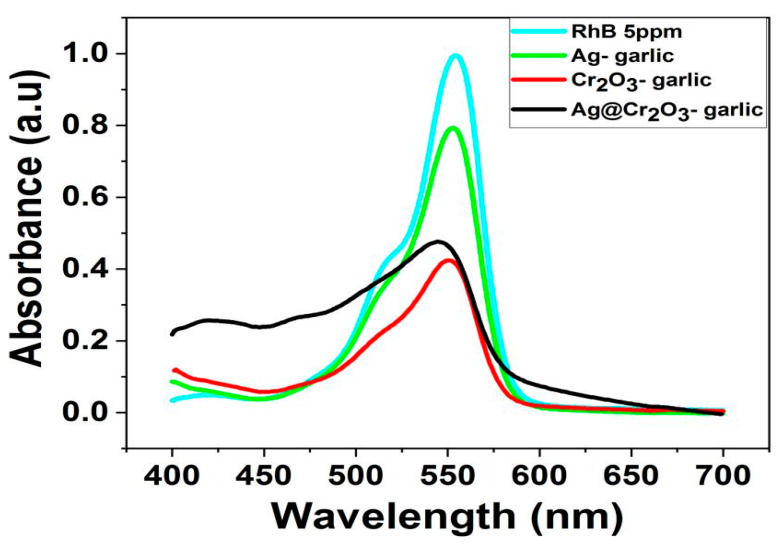
Comparison of absorption spectra of RhB without and with different catalysts.

To find out the affinity of the synthesized nanoparticles as photocatalysts, 0.02 g of each material was mixed with 50 mL of RhB (5 ppm) under sunlight (40 × 10^3^ LUX). After 30 min of treatment, 3 mL of the solution was taken, centrifuged to separate the catalyst, and subjected to a spectrophotometer to measure the RhB chromophore (λ_max_ 555 nm) [59]. As illustrated in Figure 12, the photocatalytic activity followed the order Cr_2_O_3_-garlic > Ag@Cr_2_O_3_-garlic > Ag-garlic, with degradation efficiencies of 62%, 54%, and 19%, respectively. After that, further experiments were carried out to determine the ideal parameters of Cr_2_O_3_-garlic by investigating the effects of many factors on the photocatalytic degradation of RhB, including the influence of darkness, the catalyst dose, solution pH, and contact time. The percentages of RhB that were degraded were verified using a spectrophotometer measurement of the treated solution’s maximum absorbance at 555 nm and calculated using Equation (1) [60]:

(1) $$\%\;\mathrm{RhB}\;\mathrm{degradation}=\frac{A0-At}{A0}\times 100$$

where A_0_ is the initial 5 ppm RhB absorbance (1.AU) and A_t_ is the RhB absorbance after a definite time.

#### 3.3.1. Adsorption Experiment in Darkness

**Figure 13:**
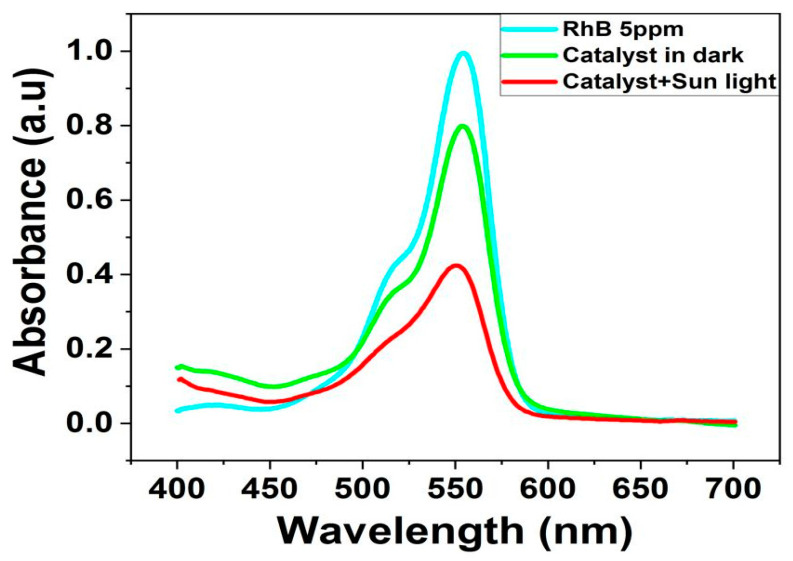
Effect of sun light on the degradation of RhB in the presence of Cr_2_O_3_-garlic.

Without a catalyst, there was no RhB degradation, as investigated by the photolysis experiment conducted with only light. The findings of the adsorption experiment in the dark demonstrated a minor loss of RhB color with a degradation efficiency of only 18.5% after 30 min, suggesting that Cr_2_O_3_-garlic has a large number of active sites that enable the dye to bind to its surface, Figure 13.

#### 3.3.2. Effect of Catalyst Mass

**Figure 14:**
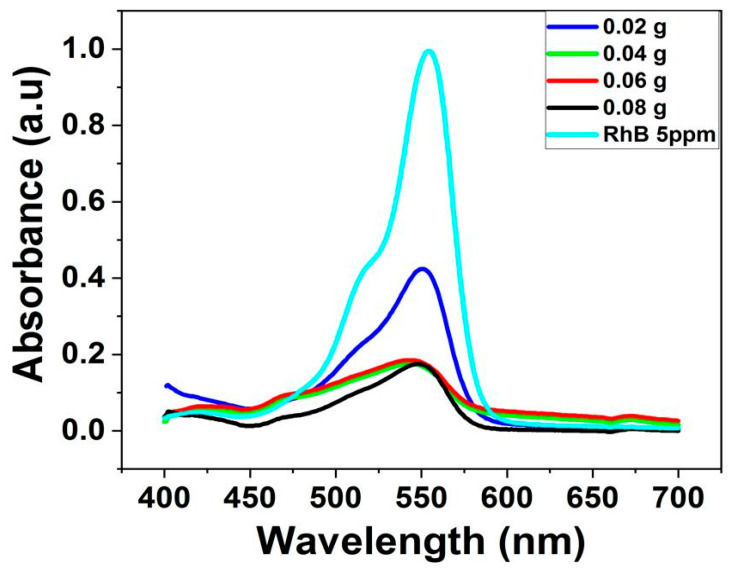
Effect of Cr_2_O_3_-garlic mass on the degradation of 5 ppm RhB (50 mL) at a natural pH.

The dosage of Cr_2_O_3_-garlic varied from 0.4 to 1.6 g/L while maintaining the contact time (30 min) and RhB concentration (5 ppm) at natural pH (7.8). The absorption spectra of RhB at various catalyst doses are shown in Figure 14. The degradation percentage increased from 62% to 88% when the catalyst dosage was increased from 0.4 g/L to 0.8 g/L because there were more fitting sites available for RhB adsorption [61]. The dose varied from 0.8 g/L to 1.6 g/L, but no discernible chromophore decrease was seen. This indicates that the best dosage for RhB molecule adsorption is 0.8 g/L for further tests.

#### 3.3.3. Effect of pH

**Figure 15:**
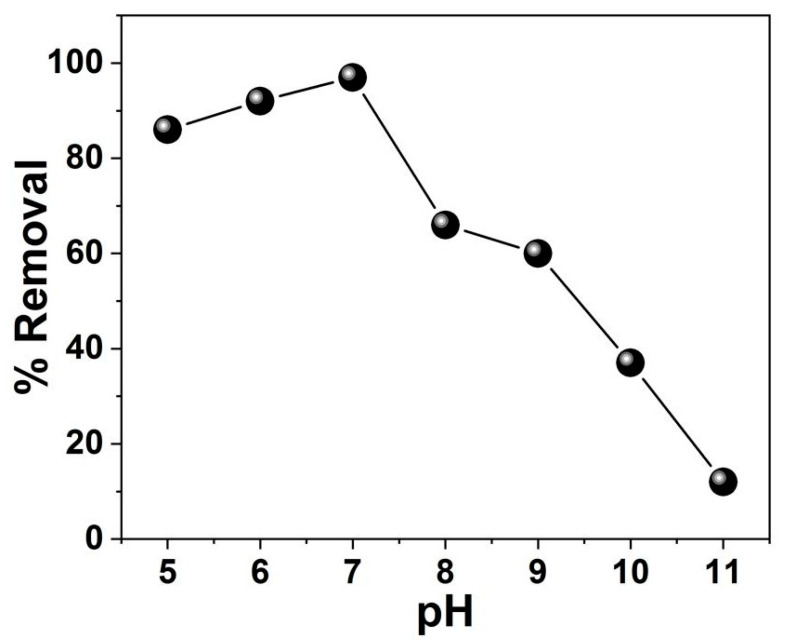
Effect of pH values on the degradation of 5 ppm RhB (50 mL) with 0.04 g of Cr_2_O_3_-garlic.

To ascertain the effects of the solution pH on photocatalytic activity, 0.04 g of Cr_2_O_3_-garlic was blended with 50 mL of RhB solution (5 ppm) for 60 min at room temperature with different pH values (5, 6, 7, 7.8 (natural), 9, 10, and 11). A solution of 0.1 N from HCl and NaOH was used to adjust the pH values of the reaction media. The greatest percentage of degradation (97.5%) occurred at pH 7, which is the ideal pH for RhB decomposition in the presence of Cr_2_O_3_-garlic as a photocatalyst (Figure 15).

#### 3.3.4. Effect of Contact Time

**Figure 16:**
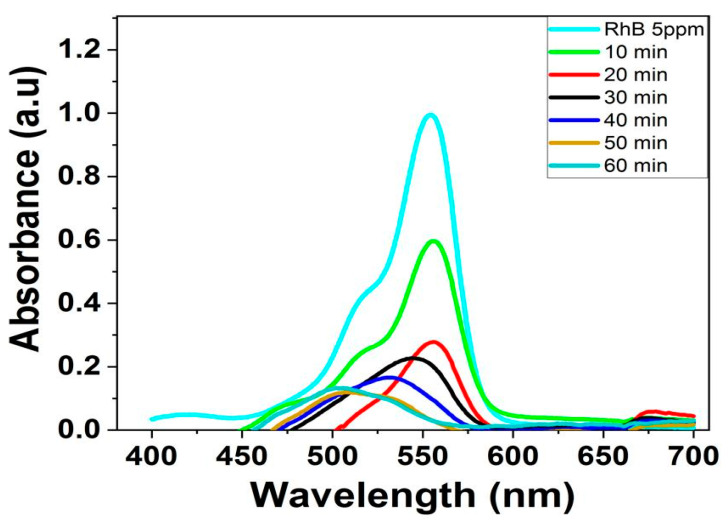
Effect of contact time on the degradation of 5 ppm RhB (50 mL) with 0.04 g of Cr_2_O_3_-garlic at pH 7.

Figure 16 shows the absorption spectra of RhB dye (5 ppm) remediation using 0.8 g/L of Cr_2_O_3_ at pH 7 at different exposure times. The percentage of eradicated RhB rose from 40% to 97% as the duration extended from 10 to 50 min. After 60 min, the degradation percentage climbed slightly to 97.5%. Due to the active sites on the Cr_2_O_3_ surface filling up and the resultant slower rate of degradation, the amount of RhB degradation increased steadily [62]. After 60 min, the maximum degradation of 97.5% was achieved.

The distinctive dye molecule absorption during the photodegradation procedure occasionally exhibits a redshift or blueshift, which may be the result of organic dyes aggregating [63]. As shown in Figure 16, RhB performed a 97.5% decolorization after 60 min; however, after 30 min to 60 min of sunlight exposure, the absorption band gradually blueshifted to below 555 nm. This indicates that the four ethyl groups in RhB are gradually eliminated until all of the ethyl groups are removed and then completely transformed into rhodamine. There was a significant absorption blueshift from 555 nm to 500 nm as a result of the deethylation of RhB molecules [64].

## 4. Conclusions

This study focused on the biosynthesis of silver nanoparticles, chromium oxide nanoparticles, and silver-decorated chromium oxide involved in the utilization of an aqueous extract obtained from garlic peel. The confirmation of the produced nanoparticles was achieved through the utilization of various physicochemical methods. Applying Rhodamine B dye (5 ppm) under solar radiation, the photocatalytic activity of nanoparticles was evaluated. Cr_2_O_3_-garlic had the highest photocatalytic effectiveness among the investigated nanoparticles, with 97.5% decomposition. An investigation was conducted to assess the antimicrobial activity against waterborne pathogens, encompassing both bacterial and fungal strains. The antimicrobial activity of Ag@Cr_2_O_3_ was the highest among the studied materials due to the synergistic effect between both Ag-Nps and Cr_2_O_3_-Nps, which improved the inhibition of microbial growth.

## Figures and Tables

**Table 1:** The effect of different nanoparticle treatments (80 ppm) on mycelial growth and the percentage of phytopathogenic fungi was inhibited.

	Diameter of Inhibition Zone (mm)				
	*A. porri*	*A. flavus*	*A. niger*	*F. oxysporum*	*T. longibrachiatum*
**Control Negative**	0	0	0	0	0
Control Positive (miconizol)	21.7 ± 0.1	24.7 ± 0.05	23.4 ± 0.23	38.7 ± 0.08	25.4 ± 0.10
GPE	13.3 ± 0.21	11.3 ± 0.32	18.9 ± 0.13	12.3 ± 0.15	9 ± 0.5
Ag-garlic	31.7 ± 0.3	31.7 ± 0.16	28.2 ± 0.21	19.6 ± 0.17	47.6 ± 0.25
Cr_2_O_3_-garlic	14.5 ± 0.24	12.3 ± 0.22	25.7 ± 0.09	12.9 ± 0.21	23.2 ± 0.22
Ag@Cr_2_O_3_-garlic	75.3 ± 0.12	77.6 ± 0.54	77.6 ± 0.13	64.7 ± 0.24	77.6 ± 0.13

**Table 2:** Antibacterial activity of the garlic peels, Ag-garlic, Cr_2_O_3_-garlic, and Ag@Cr_2_O_3_-garlic at (40 ppm) tested against *B. subtilis*, *E. faecium*, *E. coli*, and *P. aerogenosa*.

	Diameter of Inhibition Zone (mm)					
	Control Negative	Control Positive (Amoxicillin)	Garlic Peels	Ag-Garlic	Cr_2_O_3_-Garlic	Ag@Cr_2_O_3_-Garlic
*B. subtilis*	0	24 ± 0.23	06 ± 0.12	29 ± 0.26	19 ± 0.23	38 ± 0.31
*Enterococcus faecium*	0	21 ± 0.15	08 ± 0.42	27 ± 0.31	17 ± 0.33	36 ± 0.5
*E. coli*	0	25 ± 0.09	08 ± 0.25	28 ± 0.12	18 ± 0.05	39 ± 0.10
*P. aerogenosa*	0	24 ± 0.53	07 ± 0.17	26 ± 0.27	21 ± 0.12	33 ± 0.21

**Table 3:** Comparison of studied nanoparticles with similar antimicrobial studies.

Nanomaterial	Fabrication Method	Bioactivity	Effect	Reference
Cr_2_O_3_	Green method using *Abutilon indicum* extract	Antibacterial	Superior antibacterial activity against *E. coli*, *S. aureus*, *B. bronchiseptica*, and *B. subtilis*	[39]
Cr_2_O_3_	Sol–gel technique	Antibacterial	Antibacterial effectiveness against *K. pneumoniae.*	[54]
Garlic extract	Ultrasonication	Antibacterial	Potent antibacterial activity against *E. coli*, *S. aureus*, *S. mutans*, and *P. gingivalis*	[55]
Ag-NPs	Green method using garlic clove extract	Antibacterial, antibiofilm, antiparasitic and anti-breast cancer activity	Great antibacterial activity against *S. aureus* and *P. aerigunosa*	[56]
Ag-NPs	Green method using garlic extract	Antifungal	Strong potential inhibition of five strains of *Candida*.	[57]
Biosynthesis of Silver Nanoparticle	.Green method using *Allium sativum*	Antibacterial	Significant antibacterial efficacy against *B. licheniformis*, *P. aeruginosa*, and *C. auratus*	[58]
Ag@Cr_2_O_3_	Green method using garlic peel extract	Antimicrobial	Significant antimicrobial activity against *B. subtilis*, *E. faecium*, *E. coli*, *P. aeruginosa*, *A. porri*, *A. flavus*, *A. niger*, *F. oxysporum*, and *T. longibrachiatum*.	This work

## Data Availability

Data are contained within the article.
